# Alumni Association of the Veterinary Department of the New York University

**Published:** 1901-03

**Authors:** 

**Affiliations:** Secretary


### ALUMNI ASSOCIATION OF THE VETERINARY DEPARTMENT OF THE NEW YORK UNIVERSITY.

NOTICES were sent to graduates of the New York American Veterinary College for the purpose of organizing an Alumni Association of the Veterinary Department of the New York University.

The meeting was held at the college building on February 21, 1901. Dr. Eichhorn was installed as temporary Chairman and Dr. Fink as temporary Secretary. The first order of business was the nomination of officers for the new Association.

Nominations: For President, Dr. Fink; for First Vice-President, Dr. J. J. Hayes; for Second Vice-President, Dr. W. A. Young; for Secretary, Dr. Eichhorn; for Treasurer, Dr. Meiners. It was moved that the nominations be closed and the Secretary cast a ballot for each officer.

Naming of the Association: It was moved and seconded that the Association be called "Alumni Association of the Veterinary Department of the New York University."

By adoption all graduates of the New York College of Veterinary Surgeons and the American Veterinary College prior to the amalgamation of the old colleges are eligible to membership in this Association.

It was decided to adjourn and call a meeting on April 1st for the purpose of admitting new members and making arrangements to hold a joint banquet, on the same evening, of the Alumni Association of the American Veterinary College and New York College of Veterinary Surgeons and the Veterinary Department of the New York University.

A vote of thanks was tendered by the new organization to Dr. Hanson for assisting us in founding the new Alumni Association.

Moved and seconded that the meeting adjourn. DR. EICHHORN,
Secretary.

